# Electromagnetic Field as a Treatment for Cerebral Ischemic Stroke

**DOI:** 10.3389/fmolb.2021.742596

**Published:** 2021-09-07

**Authors:** Amanda Moya Gómez, Lena Pérez Font, Bert Brône, Annelies Bronckaers

**Affiliations:** ^1^ UHasselt Hasselt University, BIOMED, Diepenbeek, Belgium; ^2^ Department of Biomedical Engineering, Faculty of Telecommunications, Informatics and Biomedical Engineering, Universidad de Oriente, Santiago de Cuba, Cuba; ^3^ Centro Nacional de Electromagnetismo Aplicado, Universidad de Oriente, Santiago de Cuba, Cuba

**Keywords:** stroke, cerebral ischemia, electromagnetic field, neuroprotection, neurorehabilitation, pulsed electromagnetic field, sinusoidal electromagnetic field

## Abstract

Cerebral stroke is a leading cause of death and adult-acquired disability worldwide. To this date, treatment options are limited; hence, the search for new therapeutic approaches continues. Electromagnetic fields (EMFs) affect a wide variety of biological processes and accumulating evidence shows their potential as a treatment for ischemic stroke. Based on their characteristics, they can be divided into stationary, pulsed, and sinusoidal EMF. The aim of this review is to provide an extensive literature overview ranging from *in vitro* to even clinical studies within the field of ischemic stroke of all EMF types. A thorough comparison between EMF types and their effects is provided, as well as an overview of the signal pathways activated in cell types relevant for ischemic stroke such as neurons, microglia, astrocytes, and endothelial cells. We also discuss which steps have to be taken to improve their therapeutic efficacy in the frame of the clinical translation of this promising therapy.

## Introduction

Stroke is the most common cause of adult-acquired disability (Cichoń et al., 2017a; Urnukhsaikhan et al., 2017) and the third cause of death worldwide after heart disease and cancer (Christophe et al., 2017). Also known as cerebrovascular disease, stroke is an acute neurologic deficit that occurs when a cerebral blood vessel is ruptured or occluded, thus causing a decrease in blood supply on the brain.

When an ischemic process takes place, it triggers a series of events known as an ischemic cascade. These events initiate with hypoxia, which leads to an augmentation of cytotoxicity, hence causing inflammation, edema, and eventually neuronal death and loss of functional brain tissue. There are two major zones of neuronal injury: the core of the infarct and the penumbra. In the core zone, the blood flow is below 10–25% resulting in rapid, inevitable necrosis of neurons and supporting glial cells. This zone is surrounded by the penumbra: a rim of mild to moderately ischemic tissue in which infarction is evolving and which is viable for several hours after the insult. Many studies indicate that neurons in this zone remain functionally inactive but structurally intact and that they maintain membrane potential for several hours or more after the onset of a stroke (Strandgaard and Paulson, 1990; Xing et al., 2012; Guruswamy and ElAli, 2017). However, the longer the ischemic period lasts, the more these cells are at risk of dying. As a result, many treatments have been developed that aim to prevent the death of these neurons in the penumbra, such as recanalization therapies which recover the blood flow (Rha and Saver, 2007; Amki and Wegener, 2017).

Nowadays, tissue-plasminogen activator (tPA) is the only thrombolytic therapy for acute stroke approved for clinical use by the Food and Drug Administration (FDA) (Christophe et al., 2017). When administered intravenously within the first 4.5 h, tPA significantly improves patient outcome by approximately 30%. However, its use is limited to only a small minority of the patients, mostly due to the narrow therapeutic window of 4.5 h after stroke and multiple contra-indications (Albers et al., 2018). In addition, tPA treatment is associated with an increased risk of intra-cerebral hemorrhage (ICH) and mortality, and it requires sophisticated pre-treatment imaging (Saver et al., 2016).

A much investigated therapeutic strategy is neuroprotection. This approach aims to rescue ischemic tissue by intervention on the ischemic cascade. Although many neuroprotective agents have been shown to effectively treat ischemic stroke in animals, such as nitric oxide signal transduction downregulators, leukocyte inhibitors, phospholipid precursors, calcium channel blockers, and glutamate antagonists, clinical trials failed to demonstrate their success (Ginsberg, 2009).

**FIGURE 1:**
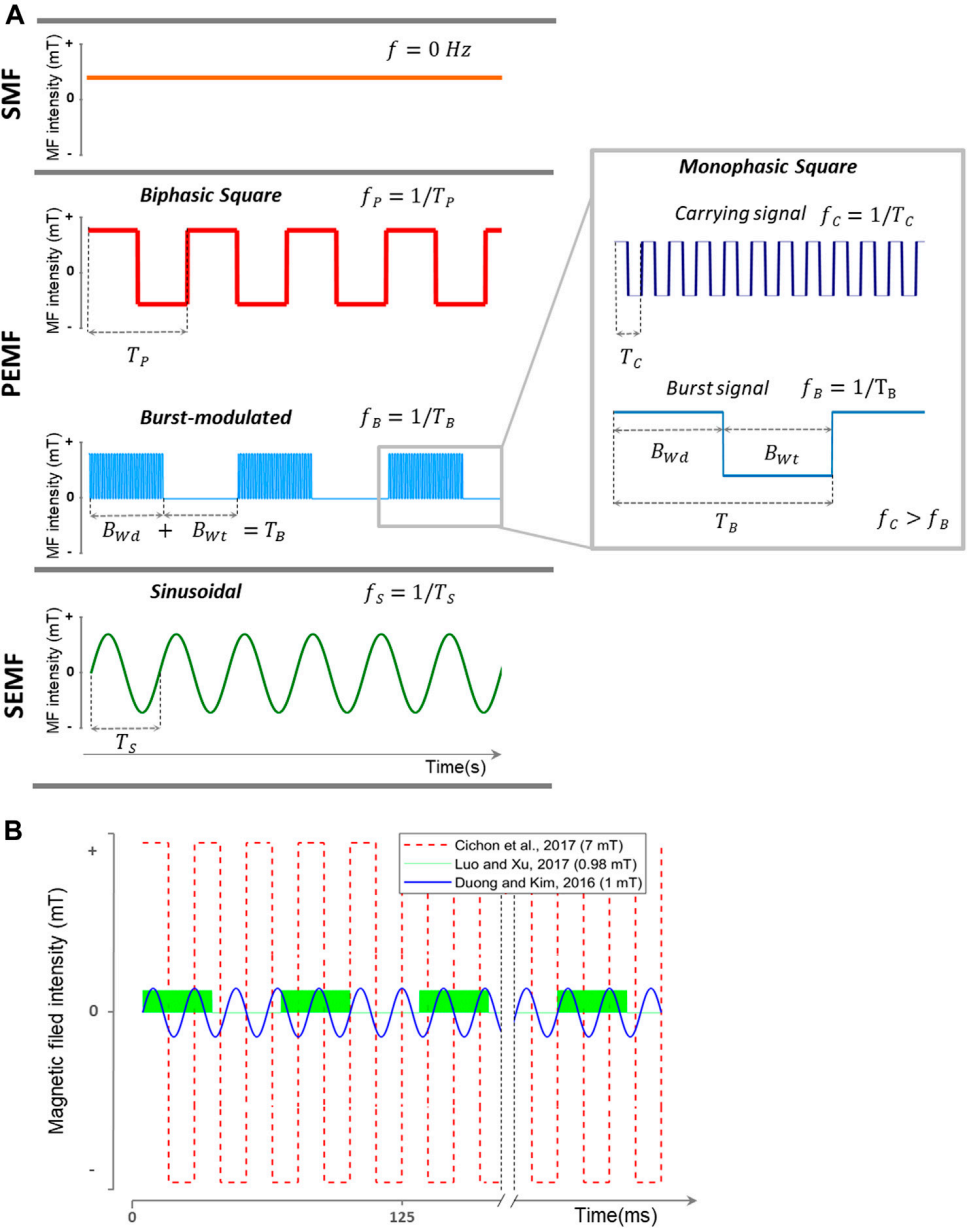
Representation of different types of EMFs used for stroke treatment. **(A)** EMF classification used in this article based on waveform and frequency (inverse of the signal period). SMF does not change its magnitude and direction in time (*f* = 0 Hz). Time-varying EMFs are divided into PEMF and SEMF. PEMFs include non-sinusoidal EMFs. This category includes monophasic square (like the carrying and burst signals), biphasic square, and burst-modulated signals. Burst-modulated signals consist of a carrying signal with a higher frequency modulated by a burst signal with a burst width and burst wait interval at a lower frequency. In this representation, *f*_*S*_ *> f*_*P*_ *> f*_*B*_. **(B)** Three of the EMF waveforms used in the consulted literature. In red (Cichoń et al., 2017a), with a 7 mT/40 Hz biphasic square PEMF. In blue (Duong and Kim, 2016), with a 1 mT/50 Hz SEMF. In green (Luo and Xu, 2017), using a burst-modulated PEMF with a burst frequency of 15 Hz (burst width, 5 ms; burst wait, 60 ms) and 4.5 MHz carrying monophasic square signal. SMF: stationary magnetic field; PEMF: pulsed electromagnetic field; SEMF: sinusoidal electromagnetic field; MF: magnetic field; *f*: frequency; *f*_*P*_: pulse frequency; *T*_*P*_: pulse period; *f*_*C*_: carrying signal frequency; *T*_*C*_: carrying signal period; *B*_*Wd*_: burst width; *B*_*Wt*_: burst wait interval; *T*_*B*_: burst period; *f*_*B*_: burst frequency; *f*_*S*_: sinusoid frequency; *T*_*S*_: sinusoid period.

Among the investigated treatment options for ischemic stroke, electromagnetic field (EMF) has been studied, first as a rehabilitation agent but later on also in the acute phase of the disease as a neuroprotective agent (Rauš et al., 2014; Cichoń et al., 2017a; Cichoń et al., 2018c). EMF is a magnetic field produced by moving electrically charged particles and can be viewed as a combination of electrical and magnetic fields. EMF can be divided into stationary magnetic fields (SMF) and time-varying magnetic fields. SMF has the same direction and magnitude with time because the electrical component is suppressed or has a 0 Hz frequency. Permanent magnets or electromagnetic coils with direct current are the most common sources of SMF. In time-varying magnetic fields, the intensity and direction of the electrical component and as a consequence the magnetic field varies over time (frequency different from 0 Hz) (Figure 1).

It has been proven that EMF affects a wide variety of biological effects. Several mechanisms of action have been proposed, including interaction with the cellular membrane, effects in calcium intracellular concentration, and free radicals production (Funk et al., 2009; Cichoń et al., 2017a). Other effects include enhancement of nitric oxide (NO) production (Sakamoto et al., 2002; Okano et al., 2005; Jelenković et al., 2006; Zulkuf Akdag et al., 2007; Cichoń et al., 2017b), increase and/or decrease of superoxide production (Selaković et al., 2013; Rauš et al., 2014; Naarala et al., 2017), and inhibition of apoptosis (Zhou et al., 2005; Palumbo et al., 2006), which are all involved in the ischemic cascade. This explains the rationale of using EMF as a stroke treatment, together with its potential to induce angiogenesis and to affect microcirculation (McKay et al., 2007; Li et al., 2015)*.* In addition, EMF has proven to reduce edema and inflammation (Morris and Skalak, 2008), while also improving neurological outcome and survival in various animal models (Rauš et al., 2012; Urnukhsaikhan et al., 2017; Font et al., 2019). A big advantage is that EMF treatment is a non-invasive method and can be applied in a patient setting, without the need for anesthesia (Shupak et al., 2003).

There are numerous studies reporting the use of EMF in stroke. However, the mode of applications (intensity, frequency, waveform, and time) is different, preclinical models vary, and the effects reported are sometimes contradictory, highlighting the need for a thorough and detailed overview. It is our goal to provide an overview of all peer-reviewed, published research articles that assessed the effect and potential mechanisms of action of EMF on cerebral stroke. This may guide future studies that aim to improve therapeutic utilization and understanding the mode of action.

## Methods

In order to identify in the primary literature all studies on EMF in cerebral stroke, a systematic search using Harzing’s Publish or Perish 5 software was made. PubMed, SCOPUS, and ISI Web of Knowledge databases were consulted. The following search terms were used: *magnetic field therapy stroke*, *magnetic field therapy ischemic stroke*, *extremely low-frequency magnetic field stroke*, *extremely low-frequency magnetic field cerebral ischemia*, *electromagnetic field therapy cerebral ischemia*, *stroke magnetic field.*

Studies using repetitive transcranial magnetic stimulation (TMS) were left out of this review because the TMS action mechanism is mostly related to neuron activation. With this technique, magnetic pulses induce an electric current in a small brain area. These pulses have, depending on TMS type, small rise times (∼0.1 ms) and higher magnetic intensity values (∼2 T) (Mishra et al., 2011). A review on the use of TMS on stroke was presented by Boonzaier et al. (2018). Only peer-reviewed, English studies using EMF as therapy in animal ischemic stroke models and patients were used in this review. Also *in vitro* experiments simulating stroke environment (oxygen and/or glucose deprivation, inflammation) using glial or neuron-like cell culture were considered. Studies presenting the use of this therapy in myocardial ischemia or other neurological diseases, or where the magnetic field was used as a diagnostic tool (magnetic resonance image) were excluded. Publications lacking specific characteristics of the applied EMF were not considered as these variables must be taken into consideration when comparing results. These exclusion criteria resulted in 27 articles included for this review, seven of which were clinical studies.

## Effect of EMFs

The first study on EMF was reported in 1990 (Rappaport and Young, 1990) evaluating the effect of EMF on focal cerebral ischemia. Since that date, 92.6% of the studies were carried out in the last decade. Most research studies involving the effect of EMFs on stroke have been focused on time-varying magnetic fields (92.6%), but its characteristics differ between studies. EMF is used in different research phases (clinical, preclinical, and *in vitro*), with only 26.0% of the studies assessed in patients. We will give an overview of the research findings in the next subsections which are organized according to EMF modality and study phase. A summary of all studies considered in this review is presented in Tables 1–6.

**TABLE 1:** Overview of *in vivo* studies using static magnetic field (SMF) as a treatment for stroke.

References	Species-stroke model	Groups (n)	SMF parameters	First exposure^a^/treatment/target	SMF effect
Bertolino et al. (2013)	Mongolian Gerbils-5 min	Healthy (12)	SMF/32 T magnetic north and south pole	Immediately after onset of ischemia/24 h × 4 days/head	↓Spontaneous motor activity
		Sh-O + Sh-E (12)			↑Time in Rota-road
	BCCAO	Ischemic (12)			↑Neural density
		Ischemic + MF (24)			
Mukhopadhyay et al. (2015)	Adults rats-15 min	Healthy	DC-EMF/30–40 mT	20 min/1 h × 10 days/not specified	↑Time in Rota-road
		Healthy + MF ischemic			↓Time to cross narrow bean walking
	MCAO	Ischemic + MF (animals per group not specified)			↑Time in the hang test
					↓Infarct size
					↑Brain activity

BCCAO: bilateral common carotid artery occlusion; MCAO: middle cerebral artery occlusion; Sh-O: sham-operated; Sh-E: sham-exposed; MF: magnetic field, DC-EMF: direct current (0 Hz) electromagnetic field.

a First exposure after the onset of ischemia.

**TABLE 2:** Overview of *in vivo* studies using pulsed electromagnetic field (PEMF) as a treatment for ischemic stroke.

References	Animal/stroke model	Groups (n)	EMF parameters	First exposure^a^/treatment/target	EMF effect
Rappaport and Young (1990)	Rats/pMCAO	Healthy (8)	27.1-MHz carrier modulated by a 65 μs pulses/585 W of peak power	15 min/2 h/head	= Brain Na^+^, K^+^, and Ca^2+^ concentrations
		Healthy + EMF (8)			
		Sh-O (12)			↓Water content 4 h after ischemia
		Ischemic (16)			
		Ischemic + EMF (16)			
Grant, Cadossi and Steinberg (1994)	Rabbits/2 h LIC + PLAC + PLMC-AO	Ischemic (6)	2.8 mT/75 Hz	10 min/2 h + 4 h of reperfusion/head	↓Edema
		Ischemic + EMF (6)	IGEA stimulator		↓Neuronal damage at the most anterior coronal level
Pena-Philippides et al. (2014)	2-month-old-C57BL/6 mice/dMCAO	Ischemic + Sh-E (12)	27.12-MHz carrier modulated by a 2 ms burst repeating at 2 bursts/s (2 Hz	30–45 min/two 15 min sessions with 4 h interval × 21 days/not specified	↓Infarct size at 21 days
		Ischemic + EMF (12)	Amplitude of 3 ± 0.6 V/m		↑2 proinflammatory and 6 dual action cytokines/chemokines
			SofPulse™ signal		↓3 proinflammatory and 1 dual action cytokines/chemokines
					↓IL-1α, TNF superfamily
Urnukhsaikhan et al. (2017)	Male C57B6/photothrombotic occlusion	Ischemic (18)	10 mT/60 Hz with a burst frequency of 4 kHz for 5 ms and a repetitive rate of 60 Hz (number of repeated single pulses: 20), pulse wait: 15 ms	Within 30–40 min/6 h × 3–14 days/whole body	↓Infarct volume at 14th day
		Ischemic + EMF(18)			↑Time in Rotarod test
					= Body weight in both groups
					↓Bad, Bax, and Caspase-3 on 3rd and 14th days
					↑Bcl-xL expression genes on 3rd day
					↑BDNF, TrkB, phospho-Akt, phospho-Bad, Bcl-xL at 3rd and 14th days
					↑IL-1β, MMP9 at 24 h and 3rd day
Bates et al. (2012)	10–13-week-old-SH rats/90 min MCAO	Ischemic (4) Ischemic + EMF I (6) Ischemic + EMF II (6) Ischemic + EMF III (6)	8 mT/I−20 Hz II-20 Hz/1 Hz III-20 Hz/6–9 Hz Positive impulse signal	I-1h, 24 hII-1h, 24h/ 2-7dIII-1h, 24h/ 2-7d/10 min session/I-injured cortexII- non-njured cortexIII-injured cortex	= Behavioral test, infarct volume, glial scarring, cell death ↑Macrophage infiltration

BCCAO: bilateral common carotid artery occlusion; pMCAO: permanent middle cerebral artery occlusion; dMCAO: distal middle cerebral artery occlusion; LIC + PLAC + PLMC-AO: left internal carotid, proximal left anterior cerebral, and proximal left middle cerebral arteries occlusion; Sh-O: sham-operated; Sh-E: sham-exposed; MF: magnetic field; EMF: electromagnetic field; IL: interleukin; TNF: tumor necrosis factor; BDNF: brain derived neurotrophic factor; Bcl-xL: B-cell lymphoma-extra-large; MMP9: matrix metallopeptidase 9; Bad: Bcl-2 associated agonist of cell death; TrkB: tropomyosin receptor kinase B; Akt: protein kinase B.

a First exposure after onset of ischemia.

**TABLE 3:** Overview of *in vitro* studies using pulsed electromagnetic field (PEMF) as a treatment for ischemic stroke.

References	Cell/ischemic stimuli	Groups	EMF parameters	EMF treatment	EMF effect
Luo and Xu (2017)	HT22 (neurons)/glutamate	Control	0.92 mT, burst frequency of 15 Hz, burst width of 5 ms and a 0.2 ms pulse width	4 h PEMF, then 10 h Glu, then 4 h PEM + 14 h Glu	↑Cell viability and ↓neurotoxicity
		Glut			↑2-AG and AEA
		PEMF + Glu			↑DAGL and NAPE-PLD (only PEMF effect)
					↓MAGL and FAAH
					↑pERK mediated by CB1R
Vincenzi et al. (2017)	SH-SY5Y (neuron-like)/hypoxia (2% O_2_) PC12 (neuron-like)/hypoxia (2% O_2_)	Normoxia Hypoxia Hypoxia + PEMF	1.5 mT/75 Hz pulse duration 1.3 ms and 0.1 duty cycle IGEA stimulator device signal	Simultaneous to hypoxia for 2–48 h	↓ Cell death (6, 12, 24, 48 h) ↓Active Caspase-3 levels (12, 24, and 48 h) ↓HIF-1α levels (2, 4, 6 h) ↓ROS production (24, 48 h)
	N9 (microglia)/hypoxia (2% O_2_)				↓ ROS production
	N9 (microglia)/hypoxia (2% O_2_)	Control LPSLPS + PEMF		Simultaneous to LPS for 24 and 48 h	↓ TNF-α, IL-1β, IL-6, and IL-8 levels
Gessi et al. (2019)	PC12-NGF-differentiated (neuron-like)/hypoxia (1% O_2_)	Normoxia Hypoxia Hypoxia + PEMF Hypoxia + inhibitor Hypoxia + inhibitor + PEMF	1.5 mT/75 Hz pulse duration 1.3 ms and 0.1 duty cycle	Simultaneous to hypoxia for 0–48 h	↑Cell viability mediated by p38 (48 h)
			IGEA stimulator device signal		↑p38 phosphorylation (10, 30 min), HSP70 levels (24 h)
					↓HSP70-increase effect with p38 inhibitor (24 h)
					↑pCREB (24 h), BDNF levels (48 h)
					↓pCREB and BDNF levels with p38 inhibitor
					↓BAD (24 h) and ↑Bcl-2 (48 h)
					↑BAD and ↓ Bcl-2 with p38 inhibitor
Merighi et al. (2020)	N9 (microglia)/LPS (1 µg/ml)	Control	1.5 mT/75 Hz pulse duration 1.3 ms and 0.1 duty cycle	Simultaneous to LPS for 24 h	↓TNF-α, IL-1β mediated by JNK1/2 MAPK
		LPS	IGEA stimulator device signal		IL-6 production
		LPS + PEMF			↑TNF-α and IL-1β production with inhibitor
		LPS + PEMF + inhibitor			↓Cell invasion (6 h) and phagocytosis (4 h)
					↑JNK1/2 phosphorylation (20 min)
					↓LPS-induced ROS levels and basal ROS levels (48, 72 h)
Vincenzi et al. (2020)	1321N1 (astrocyte)/hypoxia (1% O_2_)	Normoxia	1.5 mT/75 Hz pulse duration 1.3 ms and 0.1 duty cycle IGEA stimulator device signal	Simultaneous to hypoxia for 24 h	↑Cell viability, VEGF levels (4, 8, 16, 24 h)
		Hypoxia			= EPO, TGF-β1 levels, HIF-1α (24 h)
		PEMF			
	SH-SY5Y (neuron-like)/OGD (0.1% O_2_)	Normoxia		25% ACM with PEMF for 48 h	↑Cell viability and proliferation
		OGD			

HT22: hippocampal rat neurons; PC12: pheochromocytoma; SH-SY5Y: human neuroblastoma; PEMFs: pulsed electromagnetic fields; Glu: glutamate; NGF: nerve growth factor; HSP70: heat-shock proteins of 70; BDNF: brain-derived neurotrophic factor; pCREB: phosphorylated cAMP-response element-binding protein; OGD: oxygen and glucose deprivation; LPS: lipopolysaccharide; Bcl-2: B-cell lymphoma 2; Bad: Bcl-2 associated agonist of cell death; 2-AG: 2-arachidonoylglycerol; AEA: N-arachidonylethanolamide; DAGL: diacylglycerol lipase; NAPE-PLD: N-acyl phosphatidylethanolamine phospholipase D; MAGL: monoacylglycerol lipase; FAAH: fatty acid amide hydrolase; pERK: phosphorylated extracellular signal-regulated kinases; CB1R: cannabinoid receptor 1; HIF-1α: hypoxic induced factor; TNF-α: tumor necrosis factor; IL: interleukin; ROS: reactive oxygen species; JNK1/2: c-Jun N-terminal kinase; VEGF: vascular endothelial growth factor; EPO: erythropoietin; TGF-β1: transforming growth factor-beta 1; ACM: astrocyte conditioned medium.

**TABLE 4:** Overview of *in vivo* studies using sinusoidal electromagnetic field (SEMF) as a treatment for ischemic stroke.

References	Animal/stroke model	Groups (n)	EMF parameters	First exposure^a^/treatment/target	EMF effect
Rauš et al. (2012)	3-month Mongolian gerbils/10 min BCCAO	Healthy, Sh-O, Sh-E	0.5 mT/50 Hz/relative homogenous	Immediately/24 h × 7 days/whole body	↓Ischemic induced motor activity (24, 48 h)
		SEMF			
		Ischemic			
		Ischemic + SEMF (6–8)			
Rauš et al. (2013)		Healthy (3)	0.5 mT/50 Hz/relative homogenous	Immediately/24 h × 7 days/whole body	↓Neuronal death (14th day)
		Sh-O (6), Sh-E (6)			↑Astrocytes and microglia activation (7th day)
		SEMF			
		Ischemic (9)			
		Ischemic + SEMF (9)			
Rauš et al. (2014)		Healthy (6)	0.5 mT/50 Hz/relative homogenous	Immediately/24 h × 7 days/whole body	↑NO level, O_2_^−^ production, SOD activity, and ILP levels (7th day) (only SEMF effect)
		Sh-O (6), Sh-E (6)			↓NO, O_2_^−^, SOD activity and ILP (7th and 14th days)
		SEMF (13)			
		Ischemic (12)			
		Ischemic + SEMF (13)			
Segal et al. (2016)	SD rats-90 min tMCAO	Sh-E (6)	±0.05 mT/I-3.93 Hz	2 days/2 min × 30 days/whole body	=Body weight
		SEMF treatment I (6)	II-15.72 Hz		↑Recovery in sensory-motor functional deficits (11, 19, 27, 35, 43, and 57 days)
		SEMF treatment II (6)	Homogeneous		↓Edema and right ventricle volume (7 days, 1 and 2 months)
					↑White matter integrity of corpus callosum, fornix-fimbria, and internal capsule (1 d, 1 and 2 months)
					Progenitor cells migrated to the margins of the affected areas (57th day)
Font et al. (2019)	Wistar rat (6–8 weeks)-permanent BCCAO	Sh-O (5)	I. 13.5 mT/60 Hz	Within 3 h/20 min × 4 days/head	↑Survival and neurological outcome, locomotor activity (7 d)
		Ischemic (13)	II. 13. 5 mT/10 Hz		
		SEMF 10 Hz (13)	Non-homogeneous		↓Infarct size (only 60 Hz) (7 d)
		SEMF 60 Hz (13)			↓Survival rates with NOS inhibitor (60 Hz) (7 d)
		SEMF 60 Hz + inhibitor (4–10)			↑Ischemic area with NOS inhibitor (60 Hz) (7 d)

p-FI: permanent focal ischemia; t-GI: transient global ischemia; BCCAO: bilateral common carotid artery occlusion; tMCAO: transient middle cerebral artery occlusion; SEMF: sinusoidal electromagnetic field; Sh-O: sham-operated; Sh-E: sham-exposed; NO: nitric oxide; SOD: superoxide dismutase; ILP: Index of Lipid Peroxidation; NOS: nitric oxide synthase.

a First exposure after onset of ischemia.

**TABLE 5:** Overview of *in vitro* studies using sinusoidal electromagnetic field (SEMF) as a treatment for ischemic stroke.

References	Cell/ischemic stimuli	Groups	EMF parameters	EMF treatment	EMF effect
Boland et al. (2002)	One-week-old cultured rat hippocampal cells/SNP (0.5 μM) or Fe^2+^ (0.5 μM)	No SEMF	0, 0.005, 0.5, 0.45, 2.5, 5 mT/50 Hz (continuous)	48 h (24 h before and after SNP/Fe^2+^ addition)	=Cell mortality (only SEMF effect) (all inductions)
		SEMF			
		SEMF + SNP			=Cell mortality in presence of SNP/Fe^2+^
		SEMF + Fe^2+^			
		No SEMF	0, 0.005, 0.5, 0.45, 2.5, 5 mT/50 Hz/1 min on-off cycle for 20 min every 2 h (intermittent)	48 h (24 h before and 10 h after SNP/Fe^2+^ addition)	=Cell mortality (only SEMF effect) (all inductions)
		SEMF			
		SEMF + SNP			↑Cell mortality for 2.5, 5 mT in SNP culture
		SEMF + Fe^2+^			=Cell mortality in presence Fe^2+^
Duong and Kim (2016)	HMO6 (microglia)/OGD (1% O_2_)	OGD	10, 50, 100 Hz/1 mT 50 Hz/0.01, 0.1, 1 mT	Simultaneous to hypoxia for 4 h	↑Cell survival (50Hz/1 mT) and (10 Hz/1 mT)
		OGD + EMF	Homogeneous field		↓Intracellular Ca^2+^ and ROS (50 Hz/1 mT)
Jung and Kim (2017)	Mesenchymal stem cells/OGD (1% O_2_)	Control	10 Hz/0.01, 0.1 mT	Simultaneous to hypoxia for 3 h	↑Cell survival (10 Hz/1mT)
		OGD	50 Hz/1 mT		↓Intracellular ROS/RNS and Ca^2+^ levels (10 Hz/1 mT)
		OGD + SEMF	100 Hz/1 mT		
			Homogeneous field		
Font et al. (2019)	HMEC-1 (endothelial cell) #	Control	13.5 mT/60 Hz	20 min	↑NO levels (60 Hz)
		10 Hz 1 h	13.5 mT/10 Hz		↓NO levels with NOS inhibitor (60 Hz)
		10 Hz 24 h	Non-homogeneous		
		60 Hz 1 h			
		60 Hz 24 h			
		60 Hz 24 h + inhibitor			

SEMF: sinusoidal electromagnetic fields; SNP: sodium nitroprusside; OGD: oxygen glucose deprivation; ROS: reactive oxygen species; RNS: reactive nitrogen species; HMEC-1: human immortalized microvascular endothelial cells; NOS: nitric oxide synthase. This *in vitro* study was considered because the author also performed stroke *in vivo* experiments with SEMF.

**TABLE 6:** Overview of clinical studies assessing PEMF as a treatment for ischemic stroke.

References	Patients characteristics	Total: groups (n)	PEMF parameters	Treatment/target	PEMF effect
Capone et al. (2017)	Patients within 48 h of first monohemispheric ischemic stroke/70–90 years/NIHSS> 4/no intracranial hemorrhage, previous stroke, seizure history, and contraindications for EMF exposure/life expectancy >3 months	Two different sessions times groups (3)	1.8 mT/75 Hz pulse duration 1.3 ms and 0.1 duty cycle	45 min × 5 days	No adverse events during PEMF exposure
			IGEA stimulator device signal	120 min × 5 days/above ischemic hemisphere	↑Clinical conditions (mostly for 120 min)
					↓Volume lesion (120 min exposure)
Cichoń et al. (2017a)	Post-ischemic strokes patients/50–90 years old/NIHSS> 2/no electronics or metal implants in the EMF group, no previous cerebral disease history, no significant inflammatory factors	57: Sh-E (34), PEMF (23)	7 mT/40 Hz	15 min × 10 days (5 days a week)/pelvic girdle	↑CAT and SOD activity in hemolysate
			Rectangular and bipolar pulsed signal		=Antioxidant activity in plasma
					↑Functional status, cognitive function
					↓Depression syndrome
Cichoń et al. (2017b)	Post-stroke patients with moderate stroke severity (NIHSS)/no patients with metal or electronics implants	48: Sh-E (23), PEMF (25)	5 mT/40 Hz	15 min × 20 days (5 days a week)/pelvic girdle	↑Functional status, cognitive function
			Rectangular and bipolar pulsed signal		↓Depression syndrome
					↑3-NT and nitrate/nitrite concentration in plasma
Cichoń et al. (2018a)	Post-ischemic strokes patients	57: PEMF (23), Sh-E (34)	5 mT/40 Hz	15 min × 20 days (5 days a week)/pelvic girdle	↓Carbonyl groups in plasma proteins
			Rectangular and bipolar pulsed signal		↑Thiol groups in plasma proteins hemolysates
					↓Lipid peroxidation
					↑Functional status, cognitive function
					↓Depression syndrome
Cichoń et al. (2018b)	Post-ischemic strokes patients	48: PEMF (25), Sh-E (23)	5 mT/40 Hz	15 min × 10 days (5 days a week)/pelvic girdle	↑CAT, SOD1, SOD2, GPx1, and GPx2 mRNA expression
			Rectangular and bipolar pulsed signal		
Cichoń et al. (2018c)	Post-ischemic strokes patients	48: PEMF (25), Sh-E (23)	5mT/40 Hz	15 min × 10 days (5 days a week)/pelvic girdle	↑BDNF, VEGF, HGF, SFC plasma levels
			Rectangular and bipolar pulsed signal		↑BDNF gene expression
					=SDF-1α, βNGF, and LIF plasma level
					↓Stroke related neurologic deficit, depression syndrome
					↑Functional status, cognitive function
Cichoń et al. (2019)	Post-ischemic strokes patients	48: EMF (25), Sh-E (23)	5 mT/40 Hz	15 min × 10 days (5 days a week)	↑IL-2, IL-1β plasma levels
			Rectangular and bipolar pulsed signal		↑IL-1β mRNA expression in plasma
					=Plasma level of IFNγ and TGF-β

EMF: electromagnetic field; PEMF: pulsed electromagnetic field; NIHSS: National Institutes of Health Stroke Scale; SOD: superoxide dismutase; Sh-E: sham-exposed; CAT: catalase; GPx: glutathione peroxidase; 3-NT: 3-nitrotyrosine; BDNF: brain-derived neurotrophic factor; VEGF: vascular endothelial growth factor; HGF: hepatocyte growth factor; SFC: stem cell factor; SDF-1α: stromal cell-derived factor 1; β-NGF: β-nerve growth factor; LIF: leukemia inhibitory factor; IL: interleukin; IFNγ: interferon gamma; TGF-β: transforming growth factor-beta 1.

### Stationary Magnetic Fields

Stationary magnetic fields are constant fields, which do not change in intensity or direction over time. Two of the examined 27 studies, used this type of EMF on stroke.

Bertolino et al. (2013) evaluated the effects of SMF stimulation of 32 T after 4 days of global encephalic ischemia on motor behavior and brain morphology in gerbils. The encephalic ischemia was produced by 5 min occlusion of carotids arteries. The magnetic field stimulation was produced by a neodymium helmet surgically fixed to the skull immediately after induction of the ischemia. Treated ischemic groups were divided into two groups: magnetic north or south pole helmet. They found that the ischemic group showed a reduction in neuron density in the motor cortex, striatum, and CA1 region of the hippocampus compared to control and that this reduction was reversed, in both SMF groups. In addition, the ischemic group presented an increased spontaneous motor activity in the open field test compared to control, which was again restored by SMF stimulation. This behavior has been found by other authors (Rauš et al., 2012; Balkaya et al., 2013) and is closely associated with a significant loss of hippocampal pyramidal neurons, particularly in the CA1 area (Katsuta et al., 2003; Janac et al., 2006). In addition, the ischemic group had a reduced time on the Rotarod test which was increased after SMF treatment. It is worth mentioning that Bartolino et al. used a permanent magnet to produce the magnetic field instead of an electromagnetic stimulator and the magnetic field value used is the highest reported in the literature.

A study developed by Mukhopadhyay et al. (2015) also found that SMF reduced ischemic neuronal damage. They tested a direct current (0 Hz) magnetic field between 30 and 40 mT in rats submitted to 15 min of middle cerebral artery occlusion (MCAO). Rats were placed in the magnetic bed for 1 h daily for 10 consecutive days. Exposure was initiated 20 min after the onset of ischemia and continued throughout reperfusion. As in the study of Bertolino et al. (2013), the time in the Rotarod test was decreased after ischemia and restored by EMF. The treated group also showed a decrease in the time to cross the required distance in the narrow beam walking test and an increase in the time of hanging from the grid in the hang test compared to untreated ischemic animals. Another finding in this research was an 18% decrease in infarct size in treated groups. Interestingly, measurement of EEG in different moments of the experiment showed specific changes in EEG amplitude: stroked rats showed different rhythmic activities comparing to the control group in different regions of the brain (frontoparietal, occipital, and temporal). The ischemic group showed a decrease in all rhythmic activities in frontoparietal region, which is the most affected tissue in this stroke model. Treated rats showed significant recovery towards achieving normal rhythms.

SMF has revealed positive effects as a neuroprotective agent in ischemic stroke. Both studies consider treatment in the acute phase of the disease, and with different magnetic field intensity and application strategies, the results are beneficial in global and focal cerebral ischemia. Although the efficacy of the stationary magnetic field on ischemic stroke had been shown, the underlying molecular mechanisms of action remain to be elucidated.

### Time-Varying Magnetic Fields

Most studies have been performed with a time-changing magnetic field (25 of 27 studies). With this type of magnetic field, not only the magnetic field intensity and treatment strategy (time and days of exposure) but also the frequency and waveform can be altered. Based on the analyzed literature, we divided time-changing magnetic fields into two modalities: pulsed electromagnetic field (PEMF) and sinusoidal electromagnetic field (SEMF) (Figure 1). In PEMFs, we gathered all non-sinusoidal waveform signals. This type of EMF could be asymmetric, biphasic, quasi-rectangular, or quasi-triangular in shape (Shupak et al., 2003). Burst-modulated signals are also included in this classification. Carrier signals with a specific frequency and waveform are pulsed on and off at a specific burst frequency. SEMFs, on the other hand, follow a sinusoidal waveform. Extremely low frequency (below 300 Hz) is the most used frequency in this type of waveform. Signal frequency, burst frequency, and magnetic field intensity vary between studies, but most of them indicate a beneficial effect.

#### Pulsed Electromagnetic Field: *In Vitro* and *In Vivo* Experiments

Pioneers in this field were Rappaport and Young in 1990. They analyzed the effect of PEMF with a 27.1 MHz carrier modulated by 400 Hz pulse with 65 μs burst width and 585 W of peak power, on rats submitted to permanent MCAO (Rappaport and Young, 1990). No effect of PEMF on brain concentrations of sodium, potassium, and calcium could be detected. Brain water content was also evaluated, but even though it was significantly reduced in the treated group, changes were no consistent with the electrolyte content and water reduction was not consistent in all brain regions.

The research of Grant et al. (1994) is the most cited study in this field. These authors studied the effect of PEMF exposure in a rabbit model of ischemic stroke. Animals were submitted to 2 h occlusion of the left internal carotid, proximal left anterior cerebral, and proximal left middle cerebral arteries followed by 4 h of reperfusion (transient focal ischemia). The EMF stimulation was started 10 min after the onset of ischemia and maintained during 4 h of reperfusion. They use an EMF with 2.8 mT, 75 Hz, and pulse wide of 1.3 ms (Cadossi and Marazzi, 1988). Magnetic resonance imaging studies and histological examination were performed to assess the extent of cortical edema and ischemic neuronal damage, respectively. PEMF treatment resulted in a reduction of edema of approximately 65%. Histological examination showed a decrease of ischemic neuronal damage in the neocortical and striatum regions, but in general, the differences were not statistically significant.

A modulated PEMF (27.12 MHz carrier modulated by a 2 ms burst with 2 Hz frequency and signal amplitude of 3 ± 0.6 V/m) affected cytokine/chemokine gene expression in mice submitted to dMCAO (Pena-philippides et al., 2016). PEMF downregulated three proinflammatory and one dual action cytokine/chemokine but did not have clearly anti-inflammatory downregulation at 4 days of stroke. After 7 days, a significant downregulation of 19 genes was observed, most of them encoding proinflammatory factors. IL-1α and members of the TNF superfamily were downregulated, while IL-10 and IL-11 were upregulated; this shows that PEMFs provide a stronger suppression of inflammation at later stages of stroke. MRI imaging and TTC staining showed that this type of PEMF also reduced infarct volume and edema.

In a model of focal ischemia obtained by photothrombotic occlusion, PEMF showed an effect not only in gene expression and protein levels of inflammatory mediators but also in several apoptosis indicators (Urnukhsaikhan et al., 2017). Mice were exposed to 10 mT PEMF with a pulse frequency of 4 kHz and a burst frequency of 60 Hz and 15 ms burst wide. Treatment started within 30–40 min after ischemia induction and was maintained for 6 h/day, for up to 3 and 14 days. Infarction volume was significantly reduced by PEMF 14 days after occlusion. Treated mice had significantly longer Rotarod times compared to sham-exposed mice, indicating better motoric functions. At the molecular level BDNF, TrkB, phospho-Akt, phospho-Bad, and Bcl-xL in the peri-infarcted brain area were significantly increased compared to ischemic stroke mice without LF-PEMF treatment at 3rd and 14th day. Also, Bad, Bax, and Caspase-3 decreased in treated groups on the 3rd and 14th days. These data show that PEMF decreased apoptosis. Concerning inflammation, EMF significantly decreased levels of IL-1β and MMP9 in the peri-infarct area at 24 h and 3rd day of the experiment while IL-6β levels were not altered.

In a spontaneously hypertensive rat model of focal ischemia (induced by 90 min of MCAO) on the other hand, PEMF did not improve functional or histological outcome (Bates et al., 2012). Three different groups were submitted to diverse magnetic field application strategies: all treated groups were first submitted to 10 min of 8 mT/20 Hz PEMF with 0.3 ms pulse width, 1 h, and 24 h after reperfusion. Two groups had also an additional chronic exposure (6 days), of either 1 Hz or 6–9 Hz. The authors reported no significant effect on the behavioral tests of treated groups compared to controls and that all groups showed improvement in locomotor activity as a result of time. No significant differences were found in the ischemic volume, glial scarring, cell death, and microglial activation either. However, PEMF increased macrophage infiltration restricted to the ischemic lesion site at frequencies and intensity chosen in this research. The authors claimed that the non-beneficial or detrimental effects of this therapy may be associated with the low intensity of the PMF, its frequency, or treatment period. However, other studies used lower magnetic field intensities with beneficial effects on the ischemic model (Grant et al., 1994; Rauš et al., 2012; Rauš et al., 2013; Rauš et al., 2014).

*In vitro*, the effect of PEMF on glutamate-mediated excitotoxicity was assessed in mouse hippocampal cell line HT22 (Luo and Xu, 2017). Cells were exposed to 4 h of a PEMF of 0.92 mT, burst frequency of 15 Hz, burst width of 5 ms, and a 0.2 ms pulse width with 0.02 ms pulse wait (Wang et al., 2014). The authors used three different protocols: PEMF followed by 24 h glutamate exposition, PEMF simultaneously with 20 h glutamate, and, finally, PEMF followed by 24 h glutamate treatment with a second PEMF session 10 h after starting glutamate exposure. Results showed that PEMF reduced glutamate-induced excitotoxicity in all protocols by increasing cell viability and decreasing lactate dehydrogenase (LDH) release. The third protocol presented the best results. A deeper analysis demonstrated that PEMF exerts regulation of the glutamate-induced excitotoxicity through the endocannabinoid (eCB) system, as the beneficial effects of PEMF were suppressed by inhibition of the cannabinoid receptor CB1R. PEMF also increased the production of two types of eCBs (N-arachidonylethanolamide and 2-arachidonoylglycerol) with and without glutamate stimulation. Activation of extracellular signal-regulated kinases (ERK) signaling was an essential downstream mechanism for PEMF-mediated regulation of the eCB/CB1R. Besides, PEMF affected the eCB-related enzymatic system by increasing the enzymes involved in eCBs production while decreasing the enzymes involved in eCBs degradation. In presence of glutamate, this effect was diminished since only degrading enzymes were significantly downregulated by the PEMF. This indicates that the presence of ischemic stimuli (glutamate) changes the intracellular signaling cascades and thus the effects resulting from PEMF stimulation. This study presents a novel analysis of the mechanisms of actions related to PEMF; it also presents evidence of a prophylactic effect of PEMF. Nevertheless, future studies aiming to analyze the effect of PEMF after the glutamate exposure should be considered, in order to mimic the traditional stroke-therapy time course.

A series of *in vitro* studies have been done using a PEMF of 1.5 mT, 75 Hz (Cadossi et al., 1992), focusing on different cell types and ischemic models (Vincenzi et al., 2017, 2020; Gessi et al., 2019; Merighi et al., 2020). PEMF attenuated inflammation and hypoxia-induced injury using different cell lines (human neuroblastoma-derived SH-SY5Y cells, rat pheochromocytoma PC12 cells, and N9 microglial cells) (Vincenzi et al., 2017). PEMF decreased cell death after 6, 12, 24, and 48 h of incubation in hypoxic conditions (2% O_2_). It also decreased apoptosis after hypoxia and the active caspase-3 levels after 12, 24, and 48 h of incubation in PC12 and SH-SY5Y cells. PEMF treatment also significantly reduced HIF-1α expression (25% with respect to hypoxia) in neuron-like cells and ROS production in both cell types. Microglial cells (in hypoxic conditions) also showed a decrease in ROS production after 2 and 48 h of PEMF exposure. In N9 microglial cells exposed to lipopolysaccharide (LPS, 0.1, 0.5, or 1 µg/ml), PEMF exposure (24 and 48 h) reduced the production of very important proinflammatory cytokines (TNF-α, IL-1β, IL-6, and IL-8). These results suggest a direct anti-inflammatory effect of PEMF, consistent with other *in vivo* experiments (Pena-Philippides et al., 2014; Urnukhsaikhan et al., 2017).

In a subsequent study, the protective effect of PEMF was studied in “nerve growth factor-” (NGF-) differentiated pheochromocytoma PC12 cells, injured with 48 h of hypoxia (Gessi et al., 2019). Similar to the study of Vincenzi et al., PEMF reduced cell death by 13%. These authors also found that PEMF exerted its protective effect by triggering p38 kinase phosphorylation and stimulating cytoprotective chaperone molecule HSP70. PEMF also increased CREB phosphorylation and restored BDNF basal levels. Finally, PEMF treatment decreased BAD proapoptotic regulation and increased antiapoptotic Bcl-2 molecule. The findings that the CREB/BDNF and BAD/Bcl-2 pathways are involved are in agreement with the *in vivo* studies of Urnukhsaikhan et al. (2017). Interestingly, Caspase-3 apoptotic pathways were not activated by PEMF in the PC12 cells while Urnukhsaikhan described its reduction in ischemic PEMF treated models. The study of Gessi et al. represents one of the most complete analyses of the PEMF mechanism of action in the apoptotic pathway. Similar studies should be performed in other cell types to know whether this mechanism can be generalized.

PEMF showed a protective effect on N9 microglia cells exposed to LPS-induced inflammatory conditions (Merighi et al., 2020). PEMF significantly reduced proinflammatory TNF-α, IL-1β, and IL-6 levels after LPS (1 µg/ml). Using a plethora of inhibitors, Merighi et al. found that inhibition of the c-Jun N-terminal kinase JNK1/2 reversed the protective effect mediated by PEMF on TNF-α and IL-1β production, suggesting that the protective effect of PEMF is mediated by the recruitment of JNK1/2 MAPK kinases. In the presence of LPS, PEMF significantly increased JNK1/2 phosphorylation starting from 20 min with a maximum effect at 30 min, thus confirming the role of JNK1/2 in the protective effect of PEMF. In addition, PEMF significantly reduced crucial cell functions specific to activated microglia like ROS generation (basal and LPS-stimulated), cell invasion, and phagocytosis, all essential processes involved in inflammation. It is worth mentioning that PEMF treatment by itself did not significantly affect the cell viability of microglia.

The study of Merighi et al. is in agreement with other research regarding EMF effect on inflammation and ROS production (Pena-Philippides et al., 2014; Urnukhsaikhan et al., 2017). However, some studies show that JNK1/2 activation induces an increase in proinflammatory cytokines secretion (Resnick and Fennell, 2004; Yamasaki et al., 2012). The exact role of JNK1/2 phosphorylation in ischemic conditions should be further studied to better understand its effect on cytokine production inhibition. These authors also speculated that the PEMF effect on IL-1β levels could be related to the blocking of diverse pathways, among them p38. Interestingly, exposure to the same type of EMF, in neuron-like cells under hypoxia conditions leaded to an increase of p38 kinase phosphorylation (Gessi et al., 2019). This suggests a possible differentiated effect of EMF depending on cell type. Further research of the effect of EMF in glial cells in hypoxia conditions and using primary cells instead of cell lines should be performed. Also, the use of other inflammation stimulators like IFN-γ should be considered because these proinflammatory cytokines rather than LPS are associated with central nervous system damage and disease (Gresa-Arribas et al., 2012; Liu et al., 2017; Lively and Schlichter, 2018).

Complementing the beneficial effects of PEMF induced in microglia, PEMF triggered protective actions in astrocytes (Vincenzi et al., 2020). PEMF exposure increased vascular endothelial growth factor (VEGF**)** released in 1321N1 astrocytes in a time dependent manner, with a maximum effect at 24 h (3.2-fold vs. normoxia). Furthermore, it was observed that the PEMF-mediated increase of VEGF was independent of the activation of the transcription factor HIF-1α (using a HIF-1α inhibitor chetomin). On the other hand, no effect was found on erythropoietin and “transforming grow factor-β1” (TGF-β1), which are important proteins secreted by astrocytes and which are interesting targets for stroke treatment (Dhandapani et al., 2003; Garzón et al., 2018). An interesting result from this study is that conditioned media derived from astrocytes subjected to PMEF increased viability and proliferation of SH-SY5Y cells in 48 h OGD conditions, thus suggesting the protective effect of PEMF-astrocyte culture media.

Many open questions concerning EMF stimulation and astrocytes remain. Some studies have been done showing that EMF affects viability and morphological properties (Khodarahmi et al., 2010), microvesicle motility (Golfert et al., 2001), and apoptosis (Liu et al., 2012) of normal astrocytes cell cultures subject to different types of magnetic field treatments. Therefore, this study of Vincenzi’s group represents the first one linking astrocytes, stroke, and EMF. However, it should be noticed that PEMF-stimulated astrocytes were not subjected to ischemic conditions. It seems logical to postulate that if hypoxia and EMF separately increase VEGF (Vincenzi et al., 2020), then their combined actions should lead to a greater increase in VEGF. However, previous results have demonstrated different effects of EMF in the presence or absence of an ischemic scenario (Rauš et al., 2013). In addition, the induction of VEGF by PEMF in astrocyte was HIF-1α-independent. It is important to point out that PEMF exposure did not modulate HIF-1α expression in 1321N1 astrocytes, while in SH-SY5Y neuron-like cells, it reduced the ischemia-induced HIF-1α (Vincenzi et al., 2017) suggesting a different effect, depending on the cell type. Therefore, more research on PEMF on astrocytes in ischemic conditions should be further studied.

Even though in Vincenzi’s study astrocyte cultured media did not affect viability in neuron cells under OGD conditions (Vincenzi et al., 2020), other authors have proven that OGD-astrocyte conditioned media alone could augment neuronal death (Jin et al., 2017) and decrease microglia cell death (Redzic et al., 2015). Thus, EMF experiments where culture media is collected in cell cultures subjected to OGD or other stimuli mimicking the ischemic processes should also be performed because all cell types of the neurovascular units are affected after a stroke.

#### Sinusoidal Electromagnetic Field: *In Vitro* and *In Vivo* Experiments

Boland et al. (2002) designed a study to assess the effect of 50 Hz SEMF on rat hippocampal cells in the presence or absence of two substances that induce oxidative stress: Fe^2+^ and sodium nitroprusside (SNP) which is a NO donor. Different types of SEMF were used: 0–5 mT/50 Hz (called continuous) and an intermittent form using the same signal but with a 1 min on/off cycle. In both cases, a 48 h exposure time was used. Neither type of SEMF modified basal cell mortality. Furthermore, 0–5 mT/50 Hz had no significant effect on cell mortality in the presence of SNP or Fe^2+^. Intermittent EMF did not significantly modulate cell mortality in Fe^2+^. However, intensities of 2.5 and 5 mT potentiated cell mortality triggered by SNP. This suggested that EMF at these intensities affected NO-induced oxidative stress and may increase the ability of various toxic conditions to induce cell damage. Since SEMF treatment was carried out before and after oxidative stress induction, it is unclear whether the SEMF effect is due to cell preconditioning mechanisms.

Rauš et al. (2012), Rauš et al. (2013), and Rauš et al. (2014) developed a series of studies analyzing the effects of 0.5 mT/50 Hz sinusoidal extremely low-frequency magnetic field (ELF-MF) in the behavior, survival of neuronal cells, and oxidative stress in gerbils submitted to bilateral common carotid arteries occlusion. The locomotion activity of the ischemic and treated group was higher compared to the control group; however, 7-day exposure to ELF-MF increased motor activity in healthy gerbils but decreased ischemic induced motor activity (Rauš et al., 2012). These results are similar to those obtained by Bertolino et al. (2013) using the same animal model but SMF.

In another study using the same models and treatment, ELF-MF decreased the density of damaged neurons and increased the response of glial cells (astrocytes and microglia) in the hippocampus of ischemic gerbils (Rauš et al., 2013). Activated microglia are able to release both pro- as well as anti-inflammatory mediators during ischemic stroke, depending on the time and microenvironment. During the acute phase after stroke, microglia have a negative effect on stroke outcome, while these cells are essential for tissue regeneration in the post-acute phases (Guruswamy and ElAli, 2017). Rauš et al. (2013) stated that activated microglia were observed on the 7th day of reperfusion using Iba-1 marker. In addition, a decrease in neuronal death was observed, which may be an indicator of a possible effect of ELF-MF in microglia phenotype and/or its mediators. To understand the link between the neuroprotective effects of ELF-MF and astrocytes or microglia, the effect of EMF in glial cells submitted to ischemic conditions requires further research.

Ischemic process and ELF-MF exposure can both, independently, increase oxidative stress, by means of nitric oxide (NO) and superoxide (O_2_^−^) production, lipidperoxidation (ILP) and superoxide dismutase (SOD) activity (Rauš et al., 2014). When measured 7 days after stroke, values reaching ELF-MF alone (in non-stroke animals) are even higher than those obtained in the ischemic group. However, combined with ischemia, ELF-MF showed an oxidative reducing effect. On the 14th day after reperfusion, oxidative stress parameters in the brain of these gerbils were mostly at the control levels. Other authors have shown that ELF-MF increased oxidative stress (Boland et al., 2002; Jelenković et al., 2006; Selaković et al., 2013); however, they were mostly associated with long term exposures.

Very low intensity and very low-frequency alternating electromagnetic fields were used in rats submitted to 90 min transient MCAO by Segal et al. (2016). The frequencies used in these studies were chosen according to theta and beta waves (3.93 and 15.72 Hz, respectively) and the magnetic field intensity was ±0.05 mT. In contrast to previous animal experiments, the first EMF exposure was performed 2 days after the onset of ischemia and persisted for 30 days with 2 min sessions daily. This is the largest therapeutic window used in the literature and the largest chronic evaluation of the effect of EMF. EMF had beneficial effects in increasing the recovery of sensory-motor functional deficits in treated groups as evaluated with neurological scores and forelimb placement. MRI studies demonstrated that EMF decreased edema and right ventricle volume. Fiber tracking studies showed an increase of fibers in the white matter system of fornix-fimbria and internal capsule when compared with the untreated group. In addition, immunohistochemistry showed an increase in neural generation by the presence of Dcx+ and nestin+ neural progenitor cells in treated animals. Although only three animals per group were used for MRI, histology, and fiber tracking, this study is a pioneer analyzing the effect of EMF on the white matter. However, because of the small sample size, these results should be interpreted very carefully.

ELF-MF attenuated OGD-induced cell death, calcium, and ROS intracellular levels in microglia HMO6 cell cultures (Duong and Kim, 2016). In this study, different combinations of frequencies and intensities were used (10, 50, or 100 Hz/1 mT and 50 Hz/0.01, 0.1, or 1 mT). The best results were obtained for the combination 50 Hz/1mT. A significant but less potent protective effect was also found at 10 Hz/1 mT. These authors also found that a specific inhibitor of xanthine oxidase inhibited OGD-induced ROS production and reversed the reduction in cell survival. However, no experiments were performed to know if the beneficial actions of ELF-MF are associated with this enzyme.

In a similar experiment, ELF-MF (50 Hz/1 mT) increased cell viability and decreased intracellular ROS/RNS in mesenchymal stem cells submitted to OGD conditions and 3 h ELF-MF exposure (Jung and Kim, 2017). The other combinations (10 Hz/0.01 mT, 10 Hz/0.1 mT, and 100 Hz/1 mT) did not show any protective effect. Exposure to N-acetylcysteine (NAC) and ethylenediaminetetraacetic acid (EDTA) increased the effects of ELF-MF in ROS/RNS scavenging and intracellular calcium decrease, respectively.

From both studies, we can conclude that the effect of SEMF depends on intensity and frequency. In these studies, relatively higher intensities and frequencies below 100 Hz have better effects. Also depending on the type of cell, some frequencies produce better results. Microglia respond better to 50 Hz and mesenchymal cells to 10 Hz, which could be an indicator for the possible use of combined frequencies therapies. Segal et al. (2016) also used two different frequencies targeting functional neural network by frequency rather than location, hence achieving clinical recovery and neuronal plasticity promotion.

ELF-MF improved survival, neurological output, and locomotor activity in rats with permanent bilateral common carotid artery occlusion at 24 h and 7 days after surgery (Font et al., 2019)*.* Animals were divided into three groups: ischemic control treated with SEMF of 13.5 mT/60 Hz and treated with the same magnetic field intensity but 10 Hz. All ELF-MF regimens were given in 20 min sessions for 4 days (n = 13/group). On the 7th day after surgery, neurological impairment was significantly reduced in both 10 and 60 Hz compared to the control group. Infarction volume was also reduced by more than 50% in the 60 Hz treated groups. These authors also found that injections of the general NOS blocker L-NAME reversed the increased survival rates after ELF-MF. This suggests that the beneficial effects of ELF-MF are related to NO production. 60 Hz ELF-MF treatment also significantly increased NO levels in Human immortalized microvascular endothelial cells (HMEC- 1) 24 h after the treatment. NOS inhibitor L-NMMA was able to dose-dependently inhibit the NO production resulting from the ELF-MF exposure. This study confirms that NO production is one of the key mechanisms of the beneficial effect of ELF-MF after ischemic stroke. This is in line with previous findings that showed that L-NAME reduced the angiogenic effects of endothelial cells induced by pulsed ELF-MF (Li et al., 2015). Rauš et al. (2014) also found an increase in NO levels in their experiments but associated it with oxidative stress. Specification about the isoform of the NOS which is responsible for the NO production is needed in order to clarify the real role of the NO resulting for the EMF.

At *in vitro* and *in vivo* models, only Bates et al. (2012) have reported no beneficial effects of EMF (in this case PMF) after stroke. Of course, the lack of negative results in the literature can also be caused by the fact that negative data are hardly published (so-called negative publication bias). The study of Bates et al. is the only one reporting the use of animals with a comorbidity factor (hypertension). Investigating the effect of EMF in animals with other comorbidity factors present in patients (such as age or diabetes) is highly needed to understand the clinical potential of this treatment. In addition, the effect of EMF in hemorrhagic strokes is not described in the literature. Once the cause of hemorrhage is solved, the cellular and neuronal damage is similar to ischemic stroke (Dreier et al., 2006; MacLellan et al., 2010; Naranjo et al., 2013). Therefore, EMF as a treatment strategy should also be studied in this type of stroke.

A diversity of EMF therapies is proposed in the literature, using acute and chronic exposure and the results have been most beneficial. The acute effect of EMF is the most studied in the literature, showing an interest in the research community of using the EMF as a neuroprotective agent. However, some studies use a prolonged time of exposure (24 h for 7 days) that may be difficult to propose as therapy for clinical use.

#### Clinical Experiments: Pulsed Electromagnetic Field

Clinical studies reported in this field, such as preclinical studies, present a diversity of doses and treatments, but only PEMF has reached the clinical phase.

Capone et al. (2017) performed a dose-escalation exploratory study to assess the effect of PEMF in acute ischemic stroke patients (within 48 h after the onset of the stroke). Eligible patients for the study were those older than 18 years, with first mono-hemispheric ischemic stroke, and National Institutes of Health Stroke Scale (NIHSS) score greater than 4. Exclusion criteria were acute intracranial hemorrhage; previous ischemic or hemorrhagic stroke; the history of seizure; contraindications to magnetic fields exposure life expectancy of fewer than 3 months, and other serious illness or complex disease that may confound treatment assessment.

The same PEMF’s characteristics were used as in the studies of Grant et al. (1994) and Vincenzi et al. (2017) but with a magnetic field intensity of 1.8 mT. This small clinical trial consisted of 5 EMF sessions and a 12-month follow-up. Six patients were divided into two groups (n = 3) according to the dose-escalation scheme: 45 and 120 min. Clinical evaluation was assessed immediately after EMF treatment and reported at 30, 90, and 365 days later. Improvement of the clinical conditions was observed in all patients obtaining the best results for the longer exposure time. Also, for 120 min of exposure time, the lesion volume was reduced. Another important finding of this research was the no correlation between the intensity of the field and the volume of the lesion. No adverse events were observed in any group during PEMF exposure. Parameters like respiratory and heart rate, blood pressure, pulse oximetry, and ECG signal were stable during EMF stimulation. It is important to mention that Capone et al. (2017) wanted to include a treatment of 240 min; however, no patient accepted this exposure time because it was considered too long and potentially interfering with the standard of care. This proves our previously mentioned concern on elevated exposure times in some treatments proposed in preclinical studies.

The group of Cichón developed several studies to explore the effect of PEMF regarding brain plasticity and oxidative stress in patients (Cichoń et al., 2017a; Cichoń et al., 2017b; Cichoń et al., 2018a; Cichoń et al., 2018b; Cichoń et al., 2018c; Cichoń et al., 2019). PEMF was not applied on the brain but on the pelvic girdle. 40 Hz PEMF with a rectangular and bipolar waveform was applied. This type of waveform is different from those used in the aforementioned studies where pulses are thinner and without a negative phase.

One of the studies (Cichoń et al., 2017a) used 57 post-ischemic stroke patients with moderate stroke severity. The patients were divided into two groups: EMF (n = 23) and shame PEMF exposure (n = 34) with ages between 50 and 90 years. PEMF was applied at 7mT/40 Hz for 15 min per 10 days. Both groups underwent the same 4-week rehabilitation program. Patients with a medical history of pre-stroke dementia, hemorrhagic stroke, or inflammatory factors were left out of the experiment. No drug with anti-oxidative properties was supplied to the patients. PEMF increased catalase (CAT) and superoxide dismutase (SOD) activity in hemolysate. However, low molecular weight antioxidants in plasma showed no significant difference between groups. Functional status, depression, and cognitive impairment were assessed by Bartel Index of Activities of Daily Living (ADL), Geriatric Depression Scale (GDS), and Mini-Mental State Examination (MMSE), respectively. PEMF improved functional status in about 20% and cognitive function in about 40% of the patients. PEMF also decreased depression syndrome in 60%.

In another study (Cichoń et al., 2017b) with 7 mT/40 Hz PEMF, similar clinical results were obtained. In this trial, 48 patients were enrolled; 25 of them were submitted to 15 min of PEMF during 10 sessions. Besides the functional status, cognitive impairment, and depression severity, 3-nitrotyrosine (3-NT), nitrate/nitrite estimation, and tumor necrosis factor (TNFα) were measured in plasma. Nitric oxide synthase 2 (NOS2/iNOS) was measured in whole blood samples. An increase of 3-NT in plasma was observed in 68% of the patients in the treated group versus 17% presented by the sham-exposed group. Nitrate/nitrite concentration was also elevated in the treated group. On the other hand, iNOS and TNFα did not show a significant difference between groups. The authors demonstrated that EMF increased NO generation and its metabolites improving post-strokes treatment.

The effect of PEMF (5 mT/40 Hz) in plasma oxidative stress markers was also assessed (Cichoń et al., 2018c)*.* A statistically significant decreased level of oxidative stress parameters such as thiobarbituric acid reactive substances (TBARS), thiol groups, and carbonyl groups was found in the treated patients after 10 and 20 PEMF treatments. The reduction of oxidative stress markers was significantly greater when the number of treatments was increased. Along with these biochemical markers, the clinical outcome of the patients was evaluated using the same criteria as in Cichoń et al. (2017a). PEMF-treated patients showed an improvement in their functional status. A correlation between the change in these clinical tests and the change in carbonyl groups showed a linear relationship between these parameters. Pointing this to a reduction in plasma oxidative damage markers related to the improvement in independence, cognitive functions, and degree of depression of stroke patients (Cichoń et al., 2018c).

PEMF therapy (5 mT/40 Hz) after 10 sessions also increased the expression of genes involved with the antioxidant defense system (Cichoń et al., 2018b). CAT, SOD1, SOD2, and glutathione peroxidase GPx1 and GPx4 mRNA levels were increased by more than 100% in the treated group (n = 25) with respect to the control group (n = 23) in whole blood samples. This is in accordance with the previous work of Cichoń et al. (2017a) where EMF increased levels of SOD and CAT activity in erythrocytes.

PEMF has an effect on the growth factors involved in the neuroplasticity process during the stroke (Cichoń et al., 2018a). Treatment consisting of 10 sessions of EMF increased the plasma level of BDNF and VEGF by about 200 and 50%, respectively. BDNF mRNA expression increased by 195%. Plasma level of five different cytokines was measured obtaining an increase in hepatocyte growth factor (HGF) and stem cell factor (SCF) by 35 and 25%, respectively. Stromal cell-derived factor 1 (SDF-1α), β-nerve growth factor (β-NGF), and leukemia inhibitory factor (LIF) plasma level were not significantly different between the treated and control group. In this study, the clinical status was also evaluated. As in previous studies (Cichoń et al., 2017a; Cichoń et al., 2017b; Cichoń et al., 2018c), an increase in the functional status and cognitive function and a decrease in depression syndrome and also stroke-related neurologic deficits were observed.

In a more recent study, PEMF treatment increased plasma levels of IL-1β and IL-2, as well as IL-1β mRNA expression in whole blood samples (Cichoń et al., 2019). However, INF-γ and TGF-β plasma levels remained the same. Experimental groups and treatment were the same used by Cichón et al. in previous studies (Cichoń et al., 2017b; Cichoń et al., 2018a; Cichoń et al., 2018b).

Cichón et al. suggested that the increase of NO levels obtained in their research can be associated with nNOS and/or eNOS activities, but not with iNOS expression. They also claimed that their results are consistent with evidence shown by Cho et al. (2012) who proved that EMF increased NO in rat brains. However, Cichón et al. evaluated NO in plasma, while Cho et al. (2012) measured it in the brain. Also, time exposures of EMF were different and Cho et al. did not use stroke rodents. Moreover, Rauš et al. (2014) proved that ELF-MF has a different effect on healthy and ischemic rats with an immediate EMF exposure. Exposure time, a moment of exposure, besides and EMF characteristics are important parameters that must be evaluated comparatively. However, these results are in line with the findings of Font et al. (2019) in which NO-inhibition abrogated the beneficial effects of EMF in ischemic rats.

Clinical increase of BDNF levels in plasma agreed to the findings of Urnukhsaikhan et al. (2017) in mice. BDNF is an activator of various signaling pathways involved in the regulation of neurogenesis and the survival of neurons. Lower levels of BDNF are correlated with an increased risk of stroke, worse functional outcomes, and higher mortality (Kotlega et al., 2017). This protein upregulation can improve post-stroke outcomes and the efficacy of the rehabilitation process within a physical exercise, as can be seen in Cichón et al.’s clinical assessment results. As with CAT and SOD, an increase in BDNF levels is related to gene overexpression.

On the other hand, VEGF protein not only has a pro-angiogenic action but also has a neurotrophic and neuroprotective effect, on both the central and peripheral nervous systems. VEGF generated by ependymal cells activates and enhances neuronal precursor proliferation and growth. These results are compatible with the results of Delle Monache et al. (2008). They found an increase in VEGF receptor 2 (KDR/Flk-1) in cultured umbilical human vein endothelial cells using 1 mT/50 Hz suggesting that the EMF has an angiogenic effect. This is in contrast with another publication of the same authors showing the anti-angiogenic effect of EMF at 2 mT and the same frequency (Delle Monache et al., 2013). The magnetic field intensity used by Cichoń et al. (2017a) is 5 mT, close to the inhibitory induction; however, the conditions of the experiment are not the same, cell lines instead of stroke patients and no ischemic environment in the study of Della Monache et al.

IL-2 is a proinflammatory cytokine (Nayak et al., 2012), but a dual role has been recognized by some authors (Shachar and Karin, 2013; Pena-Philippides et al., 2014). High plasma levels of this cytokine were found in patients in the first 24 h after stroke, which decreased during the next 6 days (Nayak et al., 2012). However, Cichón et al. study reported an increase of IL-2 in the treated group, coinciding with this a good clinical outcome; this result agrees with the EMF effect reported by Pena-Philippides et al. (2014) in a mouse model. On the other hand, the increase of IL-1β reported by Cichón et al. is in contrast with several other studies where a decrease was reported (Urnukhsaikhan et al., 2017; Vincenzi et al., 2017; Merighi et al., 2020). The authors refer to that a neuroprotective role of IL-1β might be attributable to the IL-1β-dependent regulation of neurotrophic factors (Cichoń et al., 2019), specifically a positive correlation between IL-1β and BDNF levels. However, in Urnukhsaikhan et al. (2017), both of these indicators’ levels were inversely proportional. It is worthy to highlight that all *in vitro* and preclinical cytokines assessments were performed in the acute, or early sub-acute (3–4 days) stroke phases, whereas the clinical studies of Cichón et al. were made 3 weeks after stroke (Pena-Philippides et al., 2014; Urnukhsaikhan et al., 2017; Varani et al., 2017; Merighi et al., 2020). Inflammation can be both harmful and protective depending on which particular stage after a stroke. While it can contribute to the expansion of the infarct, it is also responsible for infarct resolution and influences remodeling and repair (Lambertsen et al., 2019), thus further research in both acute and chronic stages of stroke is highly necessary.

The findings of Cichón et al. are a result of a PEMF applied with a pelvic griddle contrary to the study performed by Capone et al. where ELF-MF was applied on the head. In this case, Cichón et al. explained that exposure to the head of ELF-MF can affect the activation of the epileptic focus, but no reference to any study proving this is given by the authors. Moreover, Capone et al. performed an experiment to prove the safety of the ELF-MF showing that the treatment does not produce any side effect in humans when placed onto the skull (Capone et al., 2017).

The studies of Cichón et al. are the only ones that used a localized EMF exposure far from the brain, with a biochemical and molecular mechanism elucidated. The improvement obtained may be an indicator of a more systemic effect of EMF, in a rehabilitation phase, but as a neuroprotective agent, this localization may not be effective.

In conclusion, at a clinical level, only a small number of studies were found in the literature. Similar to the *in vitro* and *in vivo* experiments, researchers use different EMF characteristics. Most studies used EMF as a therapeutic agent in a chronic phase of the disease. Only one study reported a 48 h therapeutic intervention (Capone et al., 2017). No side effects are described in any experiments and a 12-month follow-up shows a long-term effect of EMF.

## Conclusion

As can be appreciated from this review, there is increasing evidence that supports the idea that therapeutic effects can be achieved from EMF in ischemic stroke. However, except for application to orthopedics (i.e., non-union fractures), there is a long way to go before this promising treatment can be accepted as a conventional medical practice. One of the reasons is that different magnetic field exposure conditions are reported in the literature. There are several combinations of EMF: the type of the wave, intensity, frequency, the technology used, and time of exposures.

In both clinical and pre-clinical studies, there is a wide variety of applied parameters. Extremely low-frequency EMF is the most used type of EMF. Both pulsed and sinusoidal waves are used in preclinical studies, but most articles applied PEMF, which also are the only type found in clinical research. In both cases, the combination of low-frequency magnetic fields and induction of microcurrents constitute a basic mechanism underlying its biological effects. The intensity of the treatment (energy of the signal) is greater in PEMF due to the amount of time that the signal is at its maximal level; however, the sinusoidal wave presents smooth periodic oscillation and different processes can be triggered with this type of wave. It is also necessary to start using a consensus nomenclature for EMF characteristics and strategies where magnetic field induction, frequency, and waveform are clear. Overall, positive effects are obtained with both SEMF and PEMF indistinctly with different magnetic field intensities and frequencies.

Comparative studies where the type of waveform is the only variable should be performed in order to clarify the differences in their effects and to maximize their clinical potential. This type of analysis was performed by Dogru et al. in the gingiva, which spotted no general difference in the use of both types of EMF (Dogru et al., 2013).

Furthermore, there is no doubt that exposure times and the type of EMF are confounding variables. The research about the effects of EMFs on stroke is relatively new and studies are scarce. As a result, limited data is available. For results to be widely accepted, more replication of the current studies by independent research groups is needed to validate the obtained results. Nowadays, there is controversy within the literature and this tends to weaken the effect of positive findings. Much of the skepticism surrounding the therapeutic action of EMF exposure could very well be a result of the uncertainty of the implicated physiological mechanisms. Additional biochemical and molecular investigations which take into consideration all possible cell interactions, as well as ischemic triggers of the central nervous system, are needed, in order to clarify the mechanism of action of EMF as a treatment of ischemic stroke. This should be done *in vitro* and *in vivo* where influences from individual variation and complexity of animals are considered.

Electromagnetic field homogeneity is another parameter to be analyzed in order to define the best approach. Among the examined articles, only four authors used a homogenous magnetic field (Rauš et al., 2014; Duong and Kim, 2016; Segal et al., 2016; Jung and Kim, 2017). This approach guarantees the same magnetic induction in all work surfaces but such induction changes once the electromagnetic field interacts with the sample. In a non-homogeneous field, an analysis of the magnetic induction performance along the work volume should be given. However, both types of EMF have beneficial effects specifically in rats and cell cultures.

It is imperative that researchers provide not only the magnetic field intensity in the work space but also its values in the sample (mostly when working with animals and patients). This requires magnetic field modeling considering the sample volume. It is also important to start using a consensus nomenclature for EMF strategy, pointing out the magnetic field intensity (provided by the stimulator and in the work volume), wave type, frequency, field line directions, and homogeneity. This is necessary not only for the reproducibility of experiments but also for the escalation of preclinical studies to clinical trials.

Heterogeneity of the analyzed studies regarding, for example, the exposure duration, the magnetic field intensity, the biological endpoint, the cell type, and the time point of investigation is substantial and makes conclusions difficult to draw. Nevertheless, the neuroprotective potential of PEMF has been confirmed in various animal models of brain ischemia in rats, gerbils, and mice while ELF-MF has been proven in rats and mice. However, there is an urgent need in animal studies evaluating EMF in combination with comorbidity factors such as diabetes and high age. In clinical studies, Capone et al. demonstrated that PEMF influenced cortical excitability and did not produce side effects in healthy volunteers (Capone et al., 2017). The studies of Cichoń also demonstrated the beneficial effects of EMF in the post-acute phase of stroke even though PEMF was not applied on the affected area but in the pelvic griddle (Cichoń et al., 2017a; Cichoń et al., 2018a; Cichoń et al., 2019).

**FIGURE 2:**
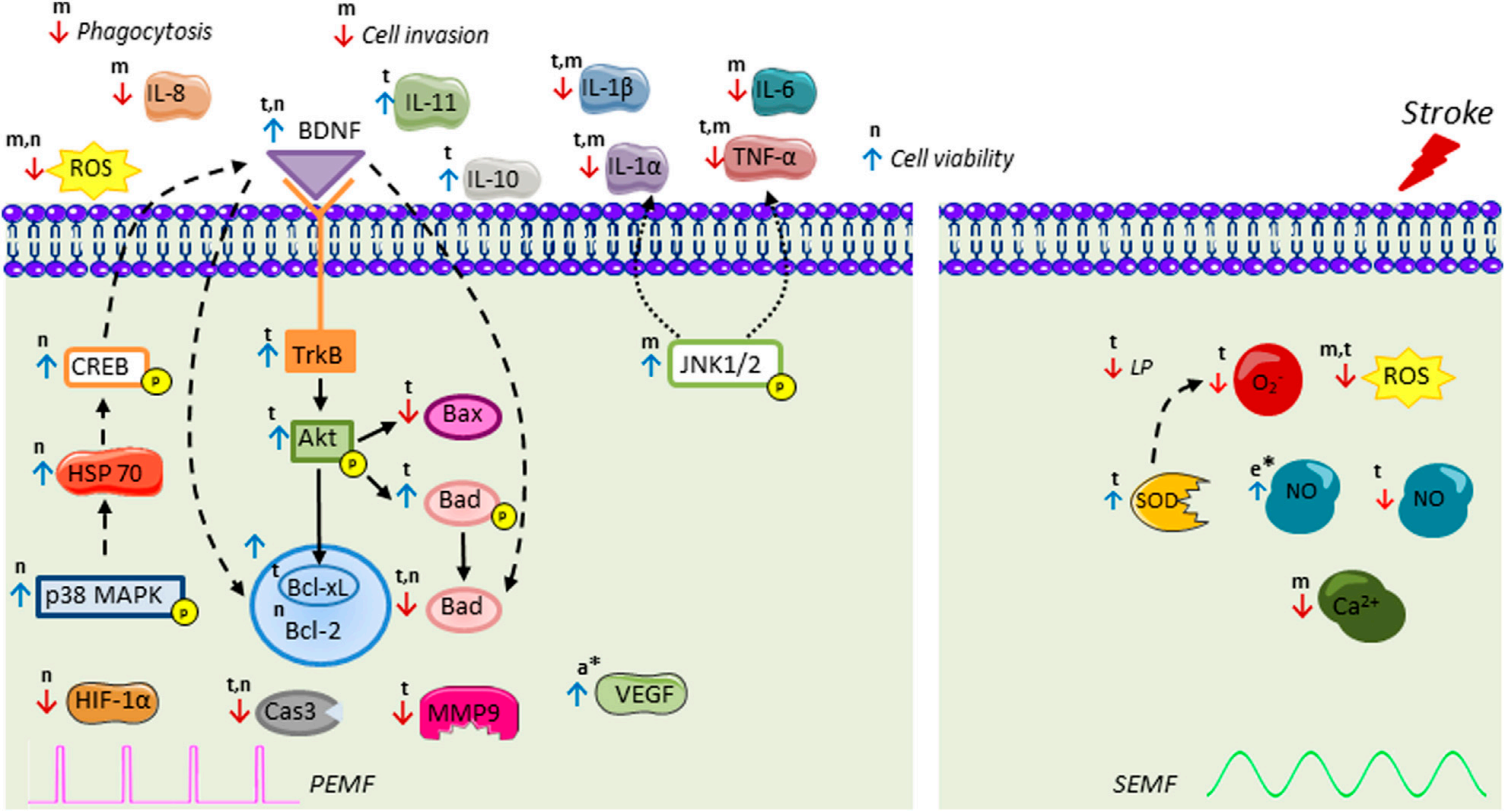
Intracellular signal transduction cascades activated by pulsed electromagnetic field (PEMF) and sinusoidal electromagnetic field (SEMF) in ischemic stroke. PEMF increases activation of p38 kinase cascade, followed by HSP70, CREB, BDNF recruitment leading to an increase of antiapoptotic Bcl-2 family and decrease in apoptotic Bad. PEMF also stimulates BDNF/TrkB/Akt pathway, thus increasing phospho-Bad that cannot bind to Bcl-xL and decreasing Bax, Bad, and Cas3. PEMF decreases MMP9, HIF-1α, ROS, and proinflammatory interleukins and upregulates anti-inflammatory IL-11 and IL-10. PEMF-induced downregulation of TNF-α and IL-1β is possibly mediated by JNK1/2. The molecular mechanisms of SEMF have been less studied. PEMF also exerts regulation on endocannabinoid system (AEA, 2-AG, MAGL, DAGL, NAPE-PLD, FAAH) with ERK signaling as an essential downstream mechanism. SEMF decreases superoxide (O_2_^−^), intracellular Ca^2+^, ROS, NO, and LP level, while it increases SOD and NO levels. Results have been assessed in t, brain tissue; n, neuron-like; m, microglia; a, astrocytes; and e, endothelial cells. * indicates only EMF stimulation without stroke. BDNF: brain-derived neurotrophic factor; CREB: cAMP response element-binding protein; HSP70: heat-shock proteins of 70; Akt: protein kinase B; TrkB: tropomyosin-related kinase B; ROS: reactive oxygen species; HIF-1: hypoxia-induced factor 1; Cas3: Caspase-3; MMP9: matrix metalloproteinase 9; IL: interleukin; TNF-α: tumor necrosis factor α; JNK1/2: c-Jun-N terminal kinase; SOD: superoxide dismutase; NO: nitric oxide; LP: lipid peroxidation; VEGF: vascular endothelial grow factor; AEA: N-arachidonylethanolamide; 2-AG: 2-arachidonoylglycerol; DAGL: diacylglycerol lipase; MAGL: monoacylglycerol lipase; NAPE-PLD: N-acyl phosphatidylethanolamine phospholipase; FAAH: fatty acid amide hydrolase. Figure was created using Servier Medical Art (smart.servier.com).

In addition, several *in vivo* and *in vitro* studies have elucidated that EMF treatment affects different processes of the ischemic cascade (Figure 2), which gives it an advantage over other studied therapies. EMF possibly activates anti-inflammatory processes, that is, a decrease in proinflammatory and an increase in anti-inflammatory cytokines. Several studies have shown which inflammatory and apoptotic genes are affected by different PEMF types (Pena-Philippides et al., 2014; Vincenzi et al., 2017; Merighi et al., 2020). Its effects in the downregulation of proinflammatory cytokines like TNF-α, IL-1β, and IL-6 have been indistinctively reported and effects in BAD proapoptotic regulation and antiapoptotic Bcl-2 molecule were obtained *in vitro* and *in vivo*. Furthermore, its effect has been proved in many cell types of the neurovascular unit. In this context, most *in vitro* studies are focused on neurons and microglia (Vincenzi et al., 2017; Gessi et al., 2019; Merighi et al., 2020) and to a lesser extent on endothelial cells (Font et al., 2019) and astrocytes (Vincenzi et al., 2020). No studies have been reported analyzing the effect of PEMF on oligodendrocytes and pericytes which are also part of the neurovascular unit. In addition, studies on endothelial cells and astrocytes cells culture in ischemic conditions are a pendent matter. Astrocytes are the most abundant cell type in the brain; they have a multi-faceted role in cerebral parenchymal homeostasis and, along with microglia, they mediate and propagate inflammatory signals (Becerra-Calixto and Cardona-Gómez, 2017). Besides, endothelial cells play an important role in angiogenesis, and co-cultures of astrocyte with endothelial cells could allow the assessment of EMF in the blood-brain-barrier model. Furthermore, ischemic models using other ischemic stimuli such as inflammation mediators (IFN-γ), excitotoxic stimuli (glutamate), or oxidative stress (H_2_O_2_) should be further explored (Luo and Xu, 2017; Imai et al., 2020).

EMF has also an effect on intracellular molecules such as ROS, Ca^2+^, and NO. Short-term MF exposure causes mild oxidative stress (modest ROS increases and changes in antioxidant levels). Regarding NO, most studies indicated that EMF induces NO. Endothelial cells produce NO after SEMF exposure. Inhibition of NOS abrogated the beneficial effects of ELF-MF in ischemic stroke in rats (Font et al., 2019). Rauš et al. (2014) also found an increase in NO levels while in clinical studies of Chicon et al., chronic exposure of ELF resulted in increased NO levels. NO can be synthesized by three isoforms of NOS: endothelial, neuronal, and inducible NOS. In an ischemic stroke setting, NO has both neurotoxic and neuroprotection effects. eNOS activation is neuroprotective, as NO in the endothelium can initiate vasodilation with a consequently cerebral blood flow increase in the ischemic region. nNOS and iNOS activation and associated NO levels have been shown to be detrimental after stroke. Further research is needed to establish the NOS isoforms and how they are activated by EMF.

The effect of EMF on Ca^2+^ levels is still unsolved. A decrease in intracellular Ca^2+^ could be explained by the cyclotron resonance model proposed by Liboff et al. (1987). This model establishes that, in the presence of a resonance process using a sinusoidal wave, specific ions can be mobilized. Since the ions resonance frequencies depend on their charge, mass, and the strength of the static magnetic field, biological effects are frequency- and intensity-dependent. The increase of intracellular calcium in the cell triggers the activation of ROS generating enzymes and ROS formation; consequently decreasing the calcium influx to the cell may decrease ROS formation. Rauš et al. associated the increase of NO resulting from EMF exposure with an increase of intracellular Ca^2+^, as NO production is mediated by Ca^2+^ intracellular concentration. Nevertheless, in the presence of ischemia, EMF decreased NO and ROS concentrations. Rappaport and Young (1990) found no effect of PEMF on Ca^2+^ after stroke in rats. However, Duong and Kim (2016) proved that, during hypoxia conditions, a sinusoidal EMF decreased intracellular Ca^2+^ and consequently ROS production; hence, EMF could involve a regulatory effect in Ca^2+^ concentration. Similar results were obtained in an experiment performed by Pessina et al. (2001) which showed downregulation of intracellular Ca^2+^ in astrocytomas after PEMF. Thus, the effect of EMF on Ca^2+^ concentrations should be further studied to elucidate the role of this important cation of the ischemic cascade.

There are many questions that require answers prior to the general acceptance of magnetic-field therapy as a primary treatment after an ischemic stroke rather than its use mainly as an adjunct therapy. Controlled, randomized, and double-blind studies must be used to optimize magnetic field conditions and duration of effect to obtain the best possible treatment.

In conclusion, even though the number of studies that assess the effect of EMF in stroke in humans and models is rather limited, convincing data have illustrated the beneficial effects in neuroprotection and recovery. EMFs have proven to have an effect on processes like apoptosis, inflammation, and oxygen and nitrogen reactive species production in neuron-like cells, endothelial cells, microglia, and astrocytes. Animal experiments have shown an effect on neurological recovery, infarct size, and edema. Small clinical studies have indicated the safety of the treatment and the beneficial effects in both acute and chronic phases of the stroke. EMF shows its self as a treatment with targets in several processes of the ischemic cascade. Unraveling the underlying mechanisms of action of the effect of EMF represents the main direction in this field as this could be used to further optimize the therapy.
